# Rapid morphological divergence following a human-mediated introduction: the role of drift and directional selection

**DOI:** 10.1038/s41437-020-0298-8

**Published:** 2020-02-20

**Authors:** Ashley T. Sendell-Price, Kristen C. Ruegg, Sonya. M. Clegg

**Affiliations:** 1 Department of Zoology, Edward Grey Institute of Field Ornithology, University of Oxford, Oxford, OX1 3PS UK; 2 Department of Biology, Colorado State University, Fort Collins, CO USA; 3 Center for Tropical Research, Institute of the Environment and Sustainability, University of California, Los Angeles, Los Angeles, CA USA; 4 Environmental Futures Research Institute, Griffith University, Queensland, 4111 Australia

**Keywords:** Population genetics, Adaptive radiation

## Abstract

Theory predicts that when populations are established by few individuals, random founder effects can facilitate rapid phenotypic divergence even in the absence of selective processes. However, empirical evidence from historically documented colonisations suggest that, in most cases, drift alone is not sufficient to explain the rate of morphological divergence. Here, using the human-mediated introduction of the silvereye (*Zosterops lateralis*) to French Polynesia, which represents a potentially extreme example of population founding, we reassess the potential for morphological shifts to arise via drift alone. Despite only 80 years of separation from their New Zealand ancestors, French Polynesian silvereyes displayed significant changes in body and bill size and shape, most of which could be accounted for by drift, without the need to invoke selection. However, signatures of selection at genes previously identified as candidates for bill size and body shape differences in a range of bird species, also suggests a role for selective processes in driving morphological shifts within this population. Twenty-four SNPs in our RAD-Seq dataset were also found to be strongly associated with phenotypic variation. Hence, even under population founding extremes, when it is difficult to reject drift as the sole mechanism based on rate tests of phenotypic shifts, the additional role of divergent natural selection in novel environments can be revealed at the level of the genome.

## Introduction

The speed at which divergent evolution proceeds is highly variable. However, the underlying processes driving differences in evolutionary rates are not well understood (Provine 1989; Barton 1998; Emerson et al. 2001; Price 2008; Emerson and Gillespie 2008). Instances of rapid phenotypic divergence following the establishment of new populations are often attributed to the strong selective pressures provided by novel biotic and abiotic environments (Millien 2006; Mathys and Lockwood 2011; Jensen et al. 2017). However, rapid divergence of phenotypic traits does not necessarily need to involve selection. Theory predicts that when new populations are established, the random sampling effect of drift during the founding event has the potential to facilitate rapid phenotypic divergence (Mayr 1942; Carson and Templeton 1984). Such rapid phenotypic changes could reflect the phenotypic attributes of founders themselves (Grant and Grant 1995; Berry 1998; Kliber and Eckert 2005; Baker et al. 2006) or arise via more complex means in which population founding results in the reorganisation of quantitative genetic variation, catalysing divergence (Mayr 1954; Carson and Templeton 1984). Despite this, studies that attribute phenotypic divergence in naturally established populations to drift and founder effects are rarer (e.g. Armstrong et al. 2018), than the numerous publications that invoke divergent natural selection (e.g. Clegg et al. 2002b; Balakrishnan and Edwards 2009; Illera et al. 2007; Rasner et al. 2004; Yeh 2004).

As the effects of drift are expected to be most pronounced when effective population sizes are small, recovery times are long and long-term effective population sizes are limited (Wright 1931; Nei et al. 1975; Clegg 2010), the opportunity for founder-induced phenotypic divergence following natural colonisation events may be limited. For example, in the silvereye (*Zosterops lateralis*) the absence of founder-induced morphological divergence following the natural colonisation of islands across the South Pacific may be explained by: establishment by founding flocks in excess of hundreds of individuals (Clegg et al. 2002a; Estoup and Clegg 2003), rapid population recovery within two to three generations (Clegg 2010) and occasional immigration from the source population (Clegg and Phillimore 2010). In contrast to natural colonisation events, human-mediated introductions may be more likely to produce rapid phenotypic shifts via founder-induced divergence, as the number of founding individuals are often smaller, recovery times often longer, long-term effective population sizes are often more limited than in natural colonisations, and where introductions are geographically remote from source populations, gene flow does not occur (Blackburn et al. 2009). In this study we examine the potential for the more extreme founding conditions associated with human-mediated introductions to produce rapid morphological divergence.

**Fig. 1:**
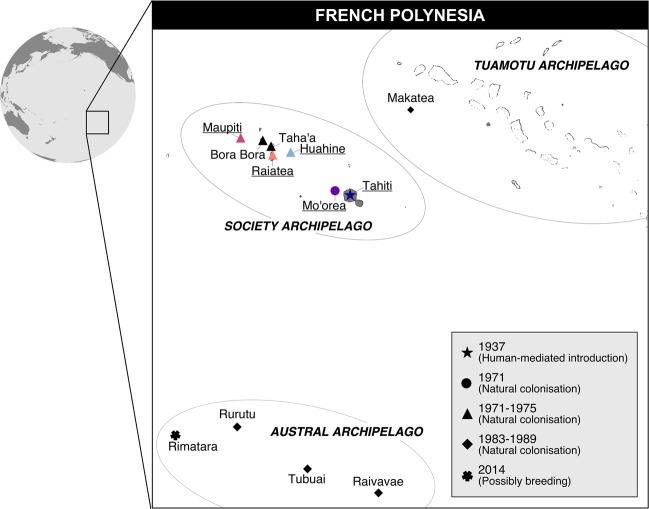
Colonisation history of the silvereye (*Zosterops lateralis*) across islands of the Austral, Society and Tuamotu archipelagos of French Polynesia following the human-mediated introduction to Tahiti in 1937. Based on distribution data in Thibault and Monnet (1990) and Thibault and Cibois (2017). Sampled islands are underlined and coloured.

The islands of French Polynesia contain a rich assemblage of introduced bird species following documented releases of at least fifty-nine species during the late 19th and early 20th centuries (Lockwood et al. 1999), with thirteen establishing breeding populations (Thibault and Cibois 2017). The silvereye was introduced from the South Island of New Zealand to the island of Tahiti in 1937 by aviculturist Eastham Guild (Guild 1938, 1940). Following introduction, the silvereye persisted in low numbers until the late 1950s after which it rapidly increased in population size and expanded into all habitat types on the island (Thibault and Monnet 1990; Monnet et al. 1993; Thibault and Cibois 2017). Following this, Tahitian silvereyes naturally dispersed to ten other island across French Polynesia, including islands within the Austral, Society and Tuamotu archipelagos (Thibault and Monnet 1990; Thibault and Cibois 2017) (Fig. 1). Although the exact number of silvereyes introduced to Tahiti is unknown, Guild’s wiritings in the Avicultural Magazine (Guild 1940), suggest the founding population likely consisted of only a handful of individuals (the individuals released were a gift from Mr G. Rowland Hutchinson, the President of the Avicultural Society of New Zealand). A small founding population size combined with a long population recovery and geographic isolation from source and neighbouring populations (the nearest silvereye population *Z. l. flaviceps* in Fiji is located over 3000 km away), provide the potential for rapid phenotypic divergence, such as changes in body/bill size and shape.

Here, we combine genome-wide tests for selection with morphological analyses to test the hypothesis that rapid phenotypic divergence can result from drift alone when populations establish under more extreme conditions than those associated with natural colonisation (very few founding individuals and slower population recovery). We address the following specific questions: (1) to what extent did the French Polynesian population experience a strong and sustained population bottleneck following introduction? (2) Can the rate of phenotypic divergence between French Polynesian and New Zealand silvereyes be explained by drift alone? and (3) What is the genomic evidence for selective processes? Commenserate with the introduction of a handful of individuals, we show that compared with natural colonisations by the silvereye, the French Polynesian population has undergone a more substantial population bottleneck followed by slower population recovery. In most instances we could not rule out drift as the sole cause of observed morphological shifts. However, the identification of outlier loci at genes previously associated with morphological divergence in birds, also suggests a strong role for directional selection.

## Materials and methods

### Sample collection

We collected blood samples and morphological data from 190 silvereyes across the islands of Tahiti, Mo’orea, Huahine, Raiatea and Maupiti (Society Archipelago) in April and May 2017. Birds were caught using mist nets and 20–40 μl of blood collected from the brachial wing vein, stored in 1 ml of lysis buffer (0.01M Tris-HCl; 0.01M NaCl; 0.01M EDTA; 1% n-lauroylsarcosine, pH 8.0) and frozen at −20 °C (Seutin et al. 1991). Following this, six morphological traits were measured. Wing length (mm) was measured as maximum flattened cord of the longest primary feather using a butted metal rule. Dial callipers (accuracy ±0.1 mm) were used to measure metatarsal length (tarsus length), mandible length to proximal edge of the nasal opening (Bill length) and mandible width and depth at the distal edge of the nasal opening (Bill width and Bill depth) (mm). Tail length (mm) of central tail feathers was measured from base to tip using either dividers or a tail rule. Wing and tail measurements were excluded from subsequent analyses if the individual was moulting. Bill length measures were excluded if the mandible was broken or overgrown. In addition, blood samples and morphological data previously collected from South Island (New Zealand) were used in the study. New Zealand samples were collected for Clegg et al. (2002a) and were collected in the same manner as French Polynesian samples. Sampling locations and the number of samples in analyses are shown in Table 1. French Polynesian birds were measured by A.T.S.P. and S.M.C., whereas New Zealand birds were measured by S.M.C. only. A comparison of morphological measurements conducted by A.T.S.P. and S.M.C. of the same six individuals showed no significant effect of data collector on any of the morphological traits measured (all two-sample *t*-test *P* values > 0.05) and therefore suggesting no strong effect attributable to different observers.

**Table 1:** Sampling information.

Island	Code	Latitude/Longitude	Morphology^a^	Sequenced^b^	Retained^c^
South Island	SNZ	−45.53/170.30	91	23	21
Tahiti	TAH	−17.58/−149.53	52	21	10
Mo’orea	MOO	−17.49/−149.83	75	21	8
Huahine	HUA	−16.82/−150.98	12	12	8
Raiatea	RAI	−16.77/−151.43	30	21	14
Maupiti	MAU	−16.45/−152.27	21	21	11

^a^Number of samples for which morphological data were collected.

^b^Number of individuals included in RAD library.

^c^Number of individuals retained in genomic analyses post-filtering.

### RAD-PE sequencing and SNP calling

For 96 French Polynesian samples, genomic DNA was extracted using Qiagen DNEasy blood and tissue extraction kits (Qiagen, Venlo, Netherlands) and restriction site-associated DNA paired-end (RAD-PE) sequencing libraries constructed at the University of California, Los Angeles following the protocol outlined in Supplementary Material. The resulting libraries were sequenced on a single Illumina HiSeq4000 lane (Illumina, San Diego, CA, USA) at the UC Davis Genome Center using paired-end 150-bp sequence reads. Following sequencing, the quality of sequencing reads was checked visually using FASTQC (Andrews 2010). Sequence reads were assigned to individuals using the *process_radtags* script in the STACKS version 1.4 software pipeline (Catchen et al. 2013). Reads containing uncalled bases and/or bases of low quality were discarded in this step using default quality thresholds (an average Phred score of 10 in sliding windows of 15% of the length of the read). Sequences with possible adaptor contamination and/or missing the *Sbf1* restriction site were also discarded. Reads were filtered to remove PCR duplicates using the STACKS *clone_filter* script. The remaining reads were then mapped to the *Zosterops lateralis melanops* genome assembly version 1 (NCBI Assembly GCA_001281735.1) (Cornetti et al. 2015) with BOWTIE2 version 2.2.6. (Langmead and Salzberg 2012) using end-to-end alignment and default settings (allowing for a maximum of two mismatches in the seed (-n 2)). Individual sample bam files were merged with existing RAD-PE sequencing reads for 23 New Zealand samples (unpublished raw data) to form a single bam file using Picard Tools version 2.7 (http://broadinstitute.github.io/picard/). Single Nucleotide Polymorphisms (SNPs) were identified using the *HaplotypeCaller* tool from the Genome Analysis Toolkit (GATK) nightly build version 2016-12-05-ga159770 (McKenna et al. 2010) and the resulting output filtered using VCFtools (Danecek et al. 2011) to remove indels and only include biallelic SNPs where the minor allele frequency > 0.01, minimum genotype quality = 30, minimum depth = 8, and where SNPs were called in at least 50% of individuals. Although more PCR duplicates would have been removed had duplicate filtering been applied following mapping of reads, given that in other widely used protocols such as double-digest RAD-sequencing (Peterson et al. 2012) duplicates cannot be removed at all, and also given that a recent study suggests PCR duplicate removal has little effect on genotyping (Euclide et al. 2020), remaining PCR duplicates are not likely to provide a substantial source of bias in our dataset.

To determine the optimum number of SNPs and individuals to retain for downstream analyses, data missingness was visualised (Fig. S1) using *genoscapeRtools* (https://github.com/eriqande/genoscapeRtools; 10.5281/zenodo.848279). The VCF file was then further filtered using VCFtools to include the optimum number of SNPs and only those individuals where less than 30% of data were missing.

As the *Z. l. melanops* genome is only assembled to the scaffold level (Cornetti et al. 2015), we mapped *Z. l. melanops* scaffolds to chromosomes of the *Taeniopygia guttata* genome assembly version 3.2.4 (NCBI Assembly GCA_000151805.2) using *Satsuma Synteny* (Grabherr et al. 2010). Output from *Satsuma Synteny* was then used to assign scaffolds to chromosomes and determine order, location and orientation using custom R scripts from Van Doren et al. (2017). Further custom scripts (see ‘Data availability’) were used to reorder the GATK outputted VCF file accordingly.

### Population structure

To investigate genetic structure among samples we conducted a Principle Component Analysis (PCA) using the full SNP dataset. We also examined patterns of population structure by performing maximum likelihood estimation of individual admixture proportions using the program *ADMIXTURE* (Alexander et al. 2009), testing *K* values 1–6. For each value of *K*, we conducted 20 independent runs and summarised runs using *CLUMPP* v.1.1.2 (Jakobsson and Rosenberg 2007). As the *ADMIXTURE* manual recommends avoiding SNPs with high linkage disequilibrium, we used the ‘–indep-pairwise 100 kb 1 0’ command in *PLINK* (Purcell et al. 2007) to remove one of every pair of SNPs with *r*^2^ > 0 within 100 kb sliding windows.

### Demographic history inference

**Fig. 2:**
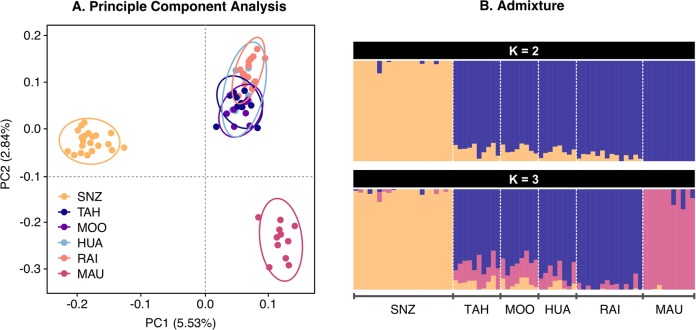
Population structure and demographic history of New Zealand (South Island) and French Polynesian silvereyes. **a** Principle component analysis of genetic variation based on 5,414 LD-filtered SNPs. The variance explained by PC1 and PC2 is 5.53% and 2.84%, respectively. **b** Maximum likelihood estimation of individual ancestries calculated with *ADMIXTURE* (Alexander et al. 2009) for all populations analysed, testing *K* values from 2 to 3 and based on 5,414 LD-filtered SNPs. Mean cross-validation error: K2 = 0.613; K3 = 0.617. SNZ South Island, New Zealand; TAH Tahiti; MOO Mo’orea; HUA Huahine; RAI Raiatea; MAU Maupiti.

**Fig. 3:**
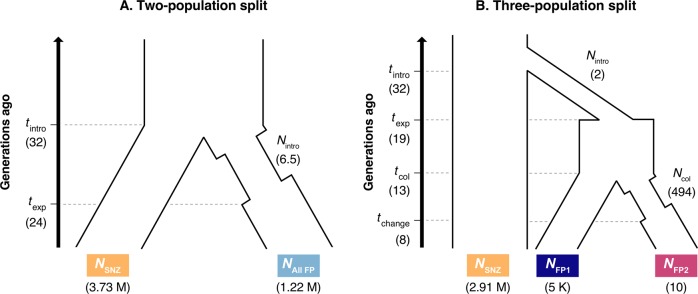
Demographic models tested with *fastsimcoal2*. **a** Two-population split where FP is treated as a single population. **b** Three-population split where FP is treated as two separate populations (FP1 = Tahiti, Mo’orea, Huahine and Raiatea; FP2 = Maupiti only). *t*_intro_ = time of introduction to Tahiti; *t*_exp_ = time of population expansion following introduction; *t*_col_ = time of colonisation of Maupiti; *t*_change_ = time of switch to long-term population size of Maupiti population; N_SNZ_ = current SNZ population size; N_All FP_ = current population size of French Polynesian population when treated as a single population; N_FP1_ = current population size of cluster 1 (Tahiti, Mo’orea, Raiatea and Huahine); N_FP2_ = current population size of cluster 2 (Maupiti); *N*_intro_ = population size following introduction; *N*_col_ = population size following colonisation of Maupiti. Values in parentheses indicate parameter estimates from best model runs.

In order to infer effective population size changes in French Polynesia, we estimated demographic parameters from the joint Site Frequency Spectrum (SFS) using the composite-likelihood and coalescent simulation approach implemented in *fastsimcoal* v. 2.6 (Excoffier and Foll 2011; Excoffier et al. 2013). As outgroup sequences were unavailable, demographic inference used the distribution of minor allele frequencies (folded-SFS). Based on findings from population structure analysis (see Fig. 2) we tested two different demographic models: a two-population demographic model which treated French Polynesia as a single population (Fig. 3a) and a three-population demographic model which incorporated a within French Polynesia population split in which Maupiti was treated as a separate population (Fig. 3b). Parameter search ranges were selected based on historical records. Assuming a generation time of 2.5 years (Kikkawa and Degnan 1998), and a known introduction time of 80 years ago, *t*_intro_ was fixed to 32 generations. Population size following introduction (*N*_intro_) was fixed between 2 and 100 individuals (within reasonable bounds for a single event, human-mediated introduction). Population recovery following introduction (*t*_exp_) was fixed between 19 and 26 generations ago (in line with timing of documented population expansion within French Polynesia). For the three-population model the establishment of the Maupiti population (*t*_col_) was fixed between 11 and 14 generations ago (in line with the documented colonisation timeframe) and founding population size of the Maupiti population (*N*_col_) fixed between 2 and 500 individuals. The three-population demographic model also incorporated a population size change within the Maupiti population (*t*_change_) which was fixed between 2 and 11 generations ago. Contemporary population sizes were estimated for each population with initial search ranges for SNZ (*N*_SNZ_), all French Polynesia (*N*_All FP_) and FP cluster 1 (*N*_FP1_) set to between 10,000 and 10,000,000 individuals. Given the very small geographic size of the island of Maupiti, search ranges for FP cluster 2 (Maupiti only) were bounded between 5 and 1,000 individuals. For each demographic model, we performed 100 independent runs (100 expectation/conditional maximisation cycles, 1,000,000 simulations per run), and chose the run with the highest likelihood as the best parameter estimates. As mutation rates for silvereyes are unknown, parameter estimates were based on estimated mutation rates for collared flycatchers (*Ficedula albicollis*): 4.6 × 10^−9^ mutations per nucleotide site per generation (Smeds et al. 2016). As *fastsimcoal* requires the use of unlinked SNPs and is sensitive to missing data, we further filtered the LD-pruned dataset (see ‘Population structure’ section) to include only SNPs present in all individuals. VCFs were then converted to SFS format using the python script *EasySFS.py* (https://github.com/isaacovercast/easySFS) using flag-a to include all sites.

### Morphological divergence

To summarise morphological divergence between the French Polynesian silvereye population(s) and representatives of its New Zealand ancestor, we conducted a PCA of morphological data collected from 189 silvereyes sampled across the Society Archipelago (French Polynesia) and 91 from Dunedin (South Island, New Zealand). PCA was conducted on log-transformed data for wing length, tail length, bill length, depth and width. Differences in PC scores between South Island and French Polynesian silvereyes were tested for significance using analysis of variance (ANOVA).

Morphological shifts between New Zealand and French Polynesia were calculated for each morphological trait measured. Morphological data were log-transformed, and shifts expressed as standard deviation units, using pooled standard deviation estimates. To determine if random processes alone could account for observed shifts in morphological traits, we calculated Lande’s (1976) statistic $N_e^ \ast$, the effective population size required to explain morphological shifts by drift alone. $N_e^ \ast$ was calculated for each morphological trait as follows:

$$N_e^ \ast = \frac{{\left( {1.96} \right)^2h^2t}}{{\left( {\frac{z}{{\upsigma }}} \right)^2}}$$

where *h*^2^ *=* narrow-sense heritability of trait in question, *t* *=* number of generations, *z* = mean morphological shift and *σ* = phenotypic standard deviation of the colonised population. Calculations of heritability (*h*^2^) were based on parent offspring, full-sibling comparisons and cross-fostering experiments in the Heron Island silvereye (*Z. l. chlorocephalus*) population. Heritability (*h*^2^) estimates for morphological traits, ranged from 0.2 and 0.6 (Clegg et al. 2002b). Based on the mean breeding age of the Heron Island silvereye, estimated from a 15-year demographic study (Kikkawa, unpublished data, as cited in Clegg et al. (2002b)), generation time was estimated at between 2 and 3 years. Using these ranges in heritability and generation time we calculated a lower estimate (2-year generation time and *h*^2^ *=* 0.2) and upper estimate (3-year generation time and *h*^2^ *=* 0.6) of $N_e^ \ast$ for each morphological trait. $N_e^ \ast$ estimates were compared with estimates of *N*_*e*_ for French Polynesia calculated as harmonic means referred to as *N*_*e*(*h*)_). The harmonic mean is always lower than the arithmetic mean and is especially sensitive to low values of *N*_*e*_. Use of the harmonic mean is therefore relevant when two related demographic scenarios are suspected, an in situ population bottleneck or a founder event (Wright 1938). Where *N*_*e*(*h*)_ > $N_e^ \ast$, drift cannot be the sole mechanism of divergence for a morphological trait and therefore selection must be invoked (Lande 1976). Both $N_e^ \ast$ and *N*_*e*(*h*)_ were calculated when treating French Polynesia as a single population (All FP), and treating French Polynesia as two separate populations (FP1 and FP2).

### Identifying signatures of selection and candidate gene analysis

To detect signatures of selection at the genomic level, we scanned for outlier loci using *PCAdapt*, a principal components-based method of outlier detection with a low rate of false-positive detection (Luu et al. 2017). *PCAdapt* requires the choice of *K* principal components, based on inspection of a scree plot, where *K* is the number of PCs with eigenvalues that depart from a straight line. *PCAdapt* then computes a test statistic based on Mahalanobis distance and controls for inflation of test statistics and false discovery rate (FDR). Outlier SNPs were identified using the following settings comparing each French Polynesian island individually to the New Zealand population: *K* = 2, MinMAF = 0.1 and FDR = 0.01. As *PCAdapt* does not included the option to assign individuals to populations, outlier detection was conducted using individual French Polynesian islands to ensure that identified outliers reflected differences between New Zealand and French Polynesia rather than within French Polynesia differences.

Known, novel or predicted genes of the *T. guttata* genome occurring within 10,000 bp of outlier SNPs were identified using the Ensembl *BioMart* database (https://www.ensembl.org/biomart). Genes that had been previously associated with morphological variation in birds were determined to be candidate genes underlying the observed divergence in body size and beak shape in silvereyes. Genes known to be involved in craniofacial variation/disease in non-avian species were also considered candidates.

### Genetic associations with morphological traits

To associated variation at SNPs with PC1 scores, we used a Bayesian sparse linear‐mixed model (BSLMM) as implemented in the software package *GEMMA* (Zhou and Stephens 2012). As BSLMM combines linear-mixed models with sparse regression models, this method is well suited to situations where the underlying genetic architecture of the trait is unknown (Zhou et al. 2013). This genome-wide association approach controls for population structure by incorporating a relatedness matrix as a covariate in the mixed model. The model was run for 20 million iterations with a burn-in of 5 million iterations. This was repeated ten times, and the resulting hyperparameters averaged across runs. As a conservative approach to identify SNPs that were significantly associated with PC1 scores, we filtered for candidate SNPs using a strict posterior inclusion probability (PIP) ≥ 0.05. This strict cut-off is five times higher than the widely used level of 0.01. *T. guttata* genes occurring within 10,000 bp of associated SNPs were identified using *BioMart*, and those previously associated with morphological variation in birds, or where a strong case could be made based on associations in other taxa, were considered candidate genes.

## Results

### RAD-PE sequencing and bioinformatics

Overall, RAD-PE sequencing resulted in an average of 227,092 paired-end reads per sample (±16,784) covering 2,128,552 variable sites. Subsequent quality filtering (removal of indels and only including biallelic SNPs where: the minor allele count was ≥2; minimum genotype quality = 30; minimum depth = 8 and SNPs were called in at least 50% of individuals) reduced the total number of SNPs to 215,543. Of the 119 samples sequenced, 72 were retained after removing individuals where ≥30% of data were missing across a subsample of 64,663 SNPs. The number of individuals retained per location ranged from 8 to 21 (Table 1). Reordering of *Zosterops* scaffolds onto *T. guttata* chromosomes, based on output from *Satsuma Synteny* and removal of unmapped/unoriented scaffolds, resulted in 63,849 SNPs (full SNP dataset). The LD-filtered dataset contained 5,414 unlinked SNPs for admixture analysis, and subsequent removal of SNPs with missing data resulted in 1587 SNPs for demographic modelling.

### Population structure

A PCA showed clear separation of individuals into three distinct clusters when plotted against PC1 and PC2 (Fig. 2a). The three clusters consisted of: (1) New Zealand only; (2) Tahiti, Mo’orea, Huahine and Raiatea combined (subsequently referred to as FP cluster 1) and (3) Maupiti only (subsequently referred to as FP cluster 2). Maximum likelihood estimation of individual ancestries calculated with ADMIXTURE (Alexander et al. 2009), consistently provided the lowest cross-validation error for *K* = 2 (mean cross-validation error across runs = 0.613) with New Zealand and French Polynesia forming distinct groups. At *K* = 3 (mean cross-validation error across runs = 0.617) admixture results were consistent with those of the PCA (Fig. 2b).

### Demographic history

Based on demographic modelling with *fastsimcoal2*, we estimated the effective population size immediately following introduction to be between 2 and 6.5 individuals, and population recovery estimated to take between 8 and 13 generations depending on the Model used. For model A (two-population split), contemporary effective population sizes for New Zealand and French Polynesia were estimated as 3.73 million and 1.22 million individuals, respectively (Fig. 3a). For model B (three-population split) effective population sizes for New Zealand, FP cluster 1 (Tahiti, Mo’orea, Huahine and Raiatea) and FP cluster 2 (Maupiti) were estimated as 2.91 million, ~5100 and 10 individuals, respectively (Fig. 3b). Based on parameter estimates, the Maupiti population (which forms its own distinct population cluster) diverged from other French Polynesia islands ~13 generations ago. The effective population size of the Maupiti population immediately following colonisation was estimated as ~494 individuals.

### Patterns and rates of morphological divergence

**Fig. 4:**
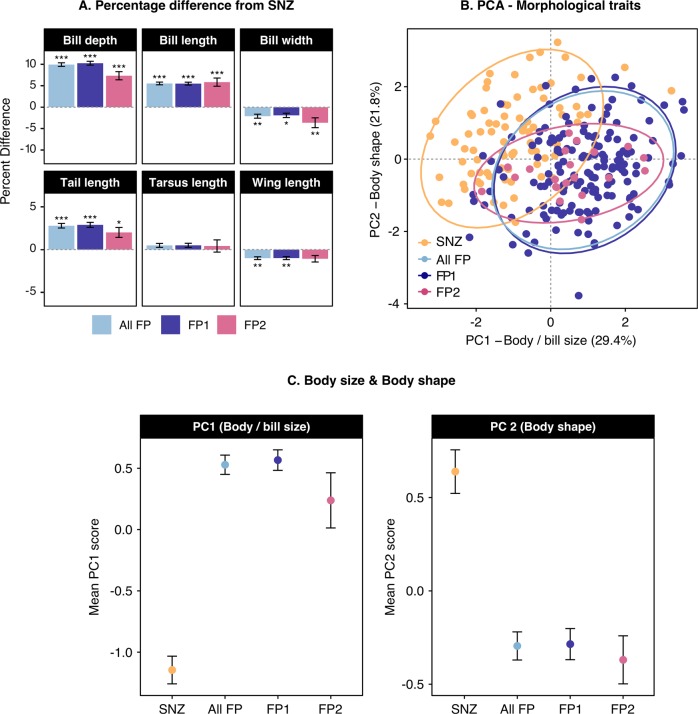
Morphological divergence of French Polynesian silvereyes. **a** The percent difference between New Zealand and French Polynesian morphological trait means. A negative percent difference in this case refers to a trait being smaller in the French Polynesian population compared with the New Zealand population, whereas a positive percent difference refers to a trait being larger in the French Polynesian population. Bars around means represent 95% confidence intervals. Asterisks indicate significant difference as determined using Wilcoxon rank sum tests: *P* *<* 0.001 (***); *P* *<* 0.01 (**); *P* *<* 0.05 (*). **b** Principal components analysis of morphological variation. Based on six morphological traits (wing length, tail length, tarsus length, bill length to posterior nasal opening and bill width/depth at anterior nasal opening) across 189 individuals of *Z. lateralis* from five islands in the Society Archipelago (French Polynesia) and 91 individuals from the South Island (New Zealand) source population. The variance explained by PC1 and PC2 is 29.4% and 21.8%, respectively (see Table S1). **c** PC1 scores (mean ± SE) and PC2 scores (mean ± SE); both calculated for New Zealand, French Polynesia combined and individual French Polynesian clusters, respectively. PC1 broadly summarises body size (all factor loadings had the same sign). PC2 broadly summarises variation in body shape (primarily contrasting bill size and body size traits).

Compared with their New Zealand ancestors, French Polynesian silvereyes had significantly longer tail lengths, longer and deeper, but narrower bills, and shorter wings (all Wilcoxon *P* values < 0.05; see Fig. 4a). Differences in tarsus length were non-significant (*W* *=* 7723.5*, P* *=* 0.189). These differences were also maintained when treating French Polynesia as two populations, with the exception wing length for FP2 (Maupiti only) which was not significantly different from New Zealand (*W* = 1192.5, *P* = 0.074; see Fig. 4a).

The first two principle components (PCs) accounted for 29.38% and 21.75% of variance, respectively. PC1 broadly summarised body/bill size (all factor loadings had the same sign, although they were not entirely even), whereas PC2 summarised body/bill shape (primarily contrasting bill size and body size traits) (see Table S1 for factor loading). Differentiation between New Zealand and French Polynesian silvereyes along PC1 and PC2 is visible in the PCA summary plot (Fig. 4b). An ANOVA indicated significant variation between population units (PC1 scores: *F*_(6,441)_ = 28.59, *P* < 0.001; PC2 scores: *F*_(6,441)_ = 10.66, *P* < 0.001). PC1 and PC2 scores differed significantly between New Zealand and French Polynesian populations, with French Polynesian silvereyes showing a trend towards larger PC1 scores (increased body/bill size) and lower PC2 scores (larger longer/deeper bills, and shorter wings) (post hoc Tukey tests, *P* < 0.001 in both cases) (Fig. 4c).

Rate tests indicated that, in most instances, drift alone could not be dismissed as the sole cause of observed morphological shifts. For example, the effective population size required to explain the observed shift in wing length when treating French Polynesia as a single population ($N_e^ \ast$ = 101–456) was well above the harmonic mean of effective population sizes at each generation (*N*_*e*(*h*)_ = 10.39). However, when using the least conservative (i.e. smallest) estimates of $N_e^ \ast$ and treating French Polynesia as a single population, drift could be rejected as the sole mechanism driving differences in bill depth and PC1 scores. The minimum effective population sizes required to explain shifts in bill length ($N_e^ \ast$ = 7) and PC1 scores ($N_e^ \ast$ = 9) were both lower than the harmonic mean of effective population sizes (*N*_*e*(*h*)_ = 10.39). When treating French Polynesia as two populations, in no case was *N*_*e*(*h*)_ > $N_e^ \ast$ (Table 2).

**Table 2:** Estimates of $N_{e}^{\ast}$, the effective population size required to explain morphological shifts by drift alone.

Trait	All FP	FP1	FP2
Wing	101–456	108–489	54–242
Tail	34.4–155	34–153	36–161
Tarsus	835–3,766	804–3,625	1,215–5,480
Bill length (P)	13–57	13–58	11–52
Bill depth (A)	7–31	7–30	7–32
Bill width (A)	192–865	241–1,086	43–195
PC1	9–38	8–37	11–51
PC2	25–113	28–126	7–32

Lower estimates are calculated with a generation time of three years and heritability estimates of 0.2, and upper estimates calculated with a generation time of 2 years and heritability estimates of 0.6. Underlined values show cases where $N_{e{(h)}}\, > \, N_{e}^{\ast}$, and drift can be rejected as the sole mechanism of differentiations.

### Signatures of selection and candidate genes

**Fig. 5:**
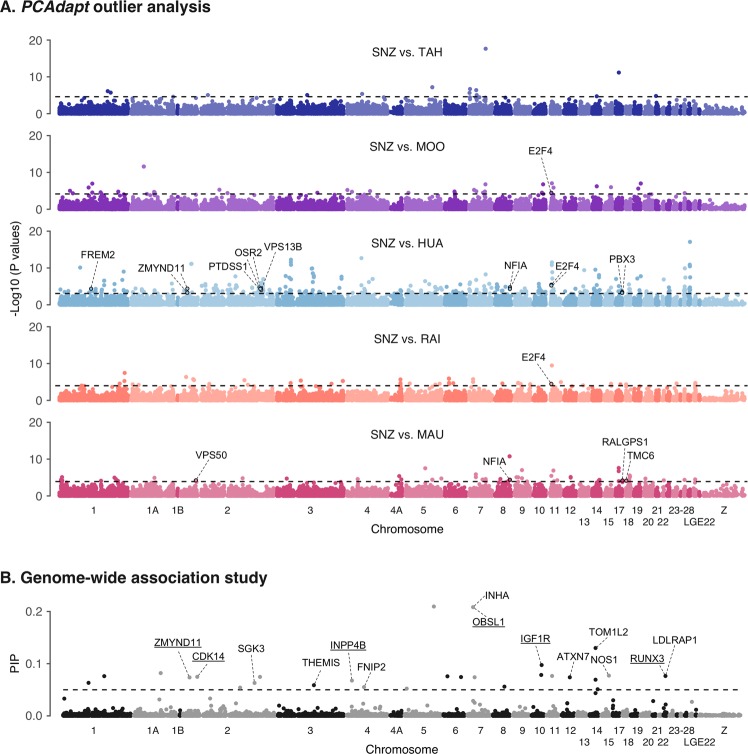
Signatures of selection in the French Polynesian silvereye population. **a** Manhattan plot of negative log10 (*P* values) estimated using *PCAdapt*. Points above the dashed line indicate outlier SNPs identified using FDR = 0.01. Genes containing outlier SNPs or within 10,000 bp of outlier SNPs and thought to be associated with morphological divergence are labelled. The distance of outlier SNPs from candidate genes is reported in Table S3. **b** Manhattan plot of BSLMM analysis of PC1 (body/bill size) scores. The dashed line indicates the threshold of posterior inclusion probability (PIP) = 0.5. Genes within 10,000 bp of SNPs with PIP ≥ 0.5 are labelled. Genes previously associated with morphological variation in birds, or where a strong case for inclusion as candidates can be made, are underlined. Chromosomes are numbered according to the *T. guttata* nomenclature. SNZ South Island, New Zealand; TAH Tahiti; MOO Mo’orea; HUA Huahine; RAI Raiatea; MAU Maupiti.

Using *PCAdapt* we identified between 15 and 509 outlier SNPs, putatively under selection (Fig. 5a). One hundred and sixty-two known, novel or predicted genes occurred within 10 kb of outlier SNPs (Table S2), of which eleven (*E2F4, FREM2, NFIA, OSR2, PBX3, PTDSS1, RALGPS1, TMC6, VPS13B, VPS50* and *ZMYND11*) have been previously associated with bill/body size differences in birds or craniofacial morphogenesis in non-avian species (Table S3). The BSLMM identified 24 SNPs with a strong association with PC1 scores (PIP ≥ 0.5) (Fig. 5b). These highly associated SNPs were located within 10,000 bp of 15 genes, of which six (*CDK14*, *OBSL1, IGF1R, INPP4B, RUNX3* and *ZMYND11*) were previously associated with morphological variation or craniofacial defects in vertebrates, including birds (Table S4). Genes not previously associated with morphological variation included: *ATXN7, FNIP2, INHA, LDLRAP1, NOS1, SGK3, THEMIS* and *TOM1L2*. A single SNP (Chr 2: 10,960,228 bp) was identified as both a *PCAdapt* outlier and strongly associated with PC1 scores in the BSLMM. This SNP was located within the *ZMYND11* (Zinc Finger MYND-Type Containing 11).

## Discussion

The introduction of the silvereye to French Polynesia provides a contemporary case in which to assess the potential for founder-induced drift to facilitate morphological divergence under more extreme founding conditions than those observed in natural colonisations by this species. Consistent with the silvereye’s capacity for rapid differentiation (Frentiu et al. 2007; Clegg et al. 2002b; Clegg et al. 2008), we observed morphological shifts of significant magnitude, and rate tests suggested these shifts could be explained solely by drift in most cases. This result demonstrates the role of stochastic effects of population founding extremes in facilitating rapid morphological shifts at the very early stage of divergence. However, a role for divergent natural selection of ecologically relevant traits was also implicated from the genomic analysis, highlighting the joint role of the two microevolutionary processes, at the early stage of divergence.

### Demographic history

While the exact date of introduction of silvereyes to French Polynesia was historically documented (Guild 1938; Thibault and Monnet 1990; Monnet et al. 1993; Thibault and Cibois 2017), Guild did not record the number of individuals he released in 1937, and as such population size immediately following founding is unknown. Combining historical records with demographic inference using the observed SFS, we were able to infer that the French Polynesian population was founded by few individuals and following introduction the population remained bottlenecked for several generations, and recovery relatively slow. This finding confirms that, compared with natural island colonisations by the silvereye which are thought to most often be established by flocks in excess of 100 individuals (Clegg et al. 2002a; Estoup and Clegg 2003) and population recovery thought to occur within 2–3 generations (Clegg 2010), the silvereye’s introduction to French Polynesia offers a more extreme case in which to assess the potential for founder-induced drift to facilitate morphological divergence. Such a small founding population size (<10 effective founders) and slow recovery (8–13 generations post introduction) is also in keeping with historical records—sightings of silvereyes were limited to around the release site until the late 1950s but becoming widespread across Tahiti by 1971 (Thibault and Cibois 2017). Although a more complete demographic model would provide further certainty regarding the demographic history of French Polynesian silvereyes, current sampling prevents this as only five of the eleven islands have been sampled. Nevertheless simple demographic models can be informative and have been used to effectively reconstruct recent known demographic histories (e.g. McCoy et al. (2014)).

### The role of drift

We observed rapid shifts in multiple morphological traits, with French Polynesian silvereyes overall showing significant increases in body and bill size and changes in body shape (longer and deeper bills, longer tails and shorter wings). Unlike previous studies of natural island colonisations by birds, in most instances we were unable to dismiss drift as the sole mechanism through which morphological shifts may have arisen using rate tests. Whereas natural island colonisations by birds may have reduced potential for founder-induced morphological divergence due to the establishment by large flocks (Vincek et al. 1997; Clegg et al. 2002a; Estoup and Clegg 2003; Illera et al. 2007), rapid population recovery following founding (Clegg 2010) and continued immigration from the source population (Clegg and Phillimore 2010), our results suggest that founder-induced morphological divergence may occur under more extreme founding conditions. This finding is at odds with other studies of avian introductions (Lima et al. 2012; Valentin et al. 2018; Gleditsch and Sperry 2019), all of which were unable to explain observed morphological shifts by drift alone despite small founding population sizes.

### Non-random founding population

While a combination of founder-induced drift and strong selective pressures associated with island-living provides the most likely explanation for morphological shifts in French Polynesian silvereyes, there is the possibility that the founding individuals were a non-random phenotypic subset of the source population. An alternative explanation for increased body size is size biased dispersal, in which only the largest of individuals survive long-range colonisation across water barriers (Clegg et al. 2002a). Parallel ‘size selective’ mechanisms could operate in human-mediated introductions; for example, Guild may have exerted size bias when releasing birds to Tahiti, or only the largest birds survived to be released. Unfortunately, such bias cannot be tested for.

### The role of natural selection

The pattern of morphological divergence we observed is broadly consistent with the island syndrome in which, amongst other repeated patterns of change, birds on islands exhibit larger body size and have more robust bills than their mainland conspecifics (Clegg and Owens 2002; Leisler and Winkler 2015). While we can only speculate on the proximate causes of selection in French Polynesia, the island syndrome is thought to extend from exposure to a common suite of biotic conditions on islands; a combination of reduced predation (Losos and Ricklefs 2009), and a shift in the balance of inter- versus intra-specific competition (Blondel 2000), features that fundamentally change selective pressures in predictable ways (Grant 1998). Given the paucity of endemic land birds on oceanic islands, the absence of competitors in French Polynesia may favour the exploitation of a greater breadth of resources, which may be facilitated by a larger body size (Grant 1968; Lack 1971). The role of interspecific competition in shaping morphology is well-evidenced (Schluter et al. 1985; Kirschel et al. 2009) and can occur remarkably rapidly, even within timescales as short as a couple of decades (Grant and Grant 2006). Alternatively, larger body size may confer an advantage in high-density island populations where aggression is expected to be increased and territoriality expected to be year-round (Kikkawa 1980a, b; Robinson-Wolrath and Owens 2003). Finally, as small size usually makes it easier to escape or hide from predators, larger body size may also result from relaxed risk of predation on islands (Lomolino 1985). Tests of these hypotheses in the Capricorn silvereye (*Z. l. chlorocephalus*) on Heron Island suggest that, in this species, increased intraspecific competition is the prevailing mechanism driving increased body size of insular forms (Kikkawa 1980b; Scott et al. 2003; Robinson-Wolrath and Owens 2003).

Despite French Polynesian silvereyes having increased PC1 scores (larger overall size) compared with their New Zealand ancestor, their wing lengths were shorter and tarsus lengths unchanged. Smaller wing size may suggest reduced reliance on flight and/or increase use of densely vegetated habitat (Savile 1957; Winkler and Leisler 1985; Leisler and Winkler 2015). However, as decreased wing length was not accompanied by an increase in tarsus length—as would be expected to aid terrestrial movement (Zeffer and Norberg 2003; Zeffer et al. 2003), this result is difficult to interpret. Interestingly, decreased wing length but no change in tarsus length has recently been reported for non-native frugivores on the Hawaiian island of O’ahu (Gleditsch and Sperry 2019).

As the observed morphological shifts have taken place within a maximum of 80 years (~32 generations), our study supports the hypothesis that the bulk of differences can develop rapidly, potentially within the first couple of hundred generations after colonisation as proposed for the Capricorn silvereye (Clegg et al. 2008). It would therefore be valuable to capture the divergence process at an even earlier stage e.g. during population establishment, as has been done for Eurasian blackbirds on the island of Heligoland (Engler et al. 2019). This may be possible in this system as the silvereye continues to expand its range in French Polynesia, for example a population has established on Rimatara (Austral archipelago) as recently as 2014 (Thibault and Cibois 2017).

### Candidate genes

Using genome-wide sequencing data we are able to identify signatures of selective processes operating within French Polynesia. Outlier SNP detection allowed us to identify 12 candidate genes potentially underlying morphological differences, eight of which were mapped to directly by SNPs putatively under directional selection and four which occurred within 10 kb of outliers. These included: *VPS50*—previously associated with bill length in Berthelot’s pipit (*Anthus berthelotii*) (Armstrong et al. 2018); *VPS13B*—identified as under directional selection between species of Darwin’s finches (Lawson and Petren 2017); *NFIA*—associated with bill length in the house sparrow (*Passer domesticus*) (Lundregan et al. 2018) and craniofacial abnormality in humans (Rao et al. 2014); *PTDSSI*—which is under directional selection in birds of paradise (Prost et al. 2018); *OSR2*—which has been experimentally demonstrated to play a role in beak development in birds (Brugmann et al. 2010) and craniofacial defects in mice (Stanier and Moore 2004); and *E2F4*, *FREM2, PBX3*, *RALGPS1, TMC6* and *ZMYND11*, which are all associated with craniofacial variation/disorders in a range of non-avian species including house mouse (*Mus musculus*), baboons (genus: *Papio*), European glass eels (*Anguilla anguilla*) and humans (Humbert et al. 2000; Cobben et al. 2014; Amaya 2015; Joganic 2016; De Meyer et al. 2017). The low overlap of genes identified across French Polynesian islands could reflect different selective pressures operating between islands, or alternatively may reflect differences in our power to detect outliers between sub-populations.

By performing a genome-wide association analysis we aimed to link phenotypic variation across French Polynesian and New Zealand silvereyes to the genomic variation in our SNP dataset. We identified several SNPs with strong associations with PC1 (body size) scores. Although not all strongly associated SNPs occurred within gene coding regions, a finding which is perhaps not surprising given the density of our marker set, several did occur within or close to gene coding regions including genes that have previously been associated with morphological variation in birds, non-avian vertebrates or implicated in craniofacial disease in humans. This included: *OBSL1*—a cytoskeletal adaptor protein previously associated with body size in humans (Hanson et al. 2009) and cetaceans (Sun et al. 2019); and *IGF1R*—a transmembrane receptor of insulin-like growth factor 1 (*IGF1*). The IGF1 pathway has a well-known role in body size scaling in a range of taxa, including chickens (Beccavin et al. 2001; Beckman et al. 2003), cattle (Grossi et al. 2015), brown house snakes (Sparkman et al. 2010) and dogs (Sutter et al. 2007); and has been previously associated with bill size in the black-bellied seedcracker (onHoldt et al. 2018). *RUNX3*—which, in the zebrafish, modulates bone morphogenetic protein (BMP) signalling during craniofacial development (Dalcq et al. 2012). BMPs such as BMP4 have been strongly associated with morphological variation of beaks in Darwin’s finches (Abzhanov et al. 2004). Finally, *CDK14*, *ZMYND11* and *INPP4B*, which have been associated with craniofacial morphology in finches, humans and house mouse, respectively (Cobben et al. 2014; Amaya 2015; Pallares et al. 2015; Lawson and Petren 2017). *ZMYND11* was the only candidate gene to be both associated with PC1 scores and also contain outlier SNPs.

In Darwin’s finches bill size is thought to be modulated by the transcriptional regulator *HMGA2:* ‘high mobility group AT-hook 2’ (Lamichhaney et al. 2015) and beak shape strongly associated with *ALX1:* ‘ALX homeobox 1’ haplotypes (Lamichhaney et al. 2016). However, as SNPs within our RAD-Seq dataset did not overlap with the position of these genes, we are unable to speculate on the role of *HMGA2* and *ALX1* in modulating bill divergence in silvereyes. Whole genome analyses would likely provide the opportunity to identify further candidate genes and allow us to address the role of *HMGA2* and *ALX1* in silvereye morphological divergence. Although not addressed here, the French Polynesian silvereye’s ability to undergo rapid morphological divergence may be further modulated by epigenetic changes. For example, in the absence of genotypic changes, epigenetic variation has been shown to facilitate rapid change in the bill size and shape of Darwin’s finches in response to sudden environmental changes such as drought or human disturbance (Skinner et al. 2014; Lamichhaney et al. 2015; McNew et al. 2017). Future work should therefore consider the role of epigenetics in facilitating rapid differentiation.

## Conclusion

Given that knowledge of contemporary natural colonisation events is limited, evolutionary insights into the early stages of divergence have been predominantly retrospective or laboratory based. However, contemporary introductions of birds to islands provide ideal systems in which to study the genomic and phenotypic changes that take place during the early stages of divergence. Taking advantage of the well-documented introduction of the silvereye to French Polynesia, we set out to assess the role of founder-induced drift in driving phenotypic divergence under more extreme founding conditions than provided by natural colonisation events in this species. While we were unable to dismiss drift as the sole cause of significant shifts in morphological traits, given that island-living is known to exert strong selective pressure on avian morphology and French Polynesian silvereyes show body size/shape changes consistent with the ‘island syndrome’, observed phenotypic shifts within the French Polynesian population likely result from a combination of drift and selective processes. Studies of colonisation in action would provide the opportunity to tease apart the roles of drift and selection in driving morphological shifts at different stages of divergence. Although, such events are extremely rare, the potential establishment of a silvereye population on the French Polynesian island Rimatara (Austral archipelago) may provide such an opportunity. Genetic and phenotypic changes associated with population founding could also be further investigated in other species introduced to French Polynesia, such as common waxbill (*Estrilda astrild*), red-browed firetail (*Neochmia temporalis*) and chestnut-breasted mannikin (*Lonchura castaneothorax*), allowing a contrast between a range of species.

## Supplementary information

Supplementary Materials

## Data Availability

Resequencing data from this study have been submitted to the European Nucleotide Archive (ENA; https://www.ebi.ac.uk/ena) under accession numbers PRJEB36361 and PRJEB25440. VCF files and custom scripts required to replicate analyses are available on dryad (10.5061/dryad.7d7wm37rm).
