# Supplementary material for: Rapid morphological divergence following a human-mediated introduction: the role of drift and directional selection
Source: Heredity (Edinb). 2020 Feb 20;124(4):535–49. doi: 10.1038/s41437-020-0298-8 (PMC7080774; doi:10.1038/s41437-020-0298-8)
Supplement: Supplementary file 1 — Supplementary Materials [file 41437_2020_298_MOESM1_ESM.docx]

**Supplementary Material**

**Rapid morphological divergence following a human-mediated introduction: The role of drift and directional selection**

***Library preparation***

100 ng of genomic DNA from each individual was digested with 2.4 U of restriction enzyme SbfI-HF (New-England Biolabs Inc., Beverly MA, USA) at 37^o^C for 60 minutes, followed by an inactivation step of 80^o^C for 20 minutes. BestRAD Sbf1 adapters were then ligated to the overhanging ends of the products of the restriction reactions by adding 50 nM to each sample with 320 U T4 DNA Ligase (New-England Biolabs Inc.). Barcoding of samples was achieved with a set of index nucleotides within the BestRAD Sbf1 adapter sequences. Reactions were incubated at 20^o^C for 16 hours and then heat-inactivated by holding at 65°C for 20 minutes. The reactions were then pooled, cleaned with AMPure XP beads, and the products randomly sheared to a mean size of 500 bp by sonication, using a BioRuptor NGS (Diagenode). Following sonication, Dynabeads M-280 streptavidin magnetic beads (Life Technologies) were used to bind the biotinylated ends of the BestRAD Sbf1 adaptors. Sbf1-HF was used once more to release adapter bound fragments and AMPure XP beads were used to clean the samples. After the bead clean up, ends were treated with Blunt End-Repair Mix found in the NEBNext Ultra DNA Library Prep Kit (New-England Biolabs Inc.) to remove overhangs. NEBNext Adaptors for Illumina sequencing were then ligated to blunt ends following a two-step incubation; first incubated at 20^o^C for 15 minutes with Blunt/TA Ligase Master Mix (New-England Biolabs Inc.) and then at 37^o^C for 15 minutes with USER Enzyme (New-England Biolabs Inc.). Following size selection using AMPure XP beads to isolate fragments within the size range 300–700 bp, the library was PCR enriched using 10 uM of P1 index primer and 10 uM of Universal PCR Primer (NEB). First a test PCR was run using 5 ul of DNA with 15 PCR cycles. Depending on the brightness of the band produced, the final PCR cycle number was adjusted from 9-12 cycles. PCR products were cleaned using AMPure XP beads. Libraries were sequenced on three Illumina HiSeq4000 lanes (Illumina, San Diego, CA, USA) at the UC Davis Genome Center using paired-end 150-bp sequence reads.

**Table S1:** Principal components analysis of morphological data. Coefficients of correlation between morphological variables and the derived principal components extracted by principal components analysis.

| **Morphological trait** | **Factor loadings** | |
| --- | --- | --- |
|  | **PC1** | **PC2** |
| Wing length | 0.265 | 0.719 |
| Tail length | 0.487 | 0.429 |
| Tarsus length | 0.297 | - |
| Culmen length (P) | 0.525 | -0.419 |
| Culmen depth (A) | 0.571 | -0.284 |
| Culmen width (A) | - | 0.182 |
| Variance explained | 29.38% | 21.75% |

**Table S2:** Known, novel or predicted genes occurring within 10kb of outlier SNPs identified, using *PCAdapt*, between New Zealand and French Polynesian islands. Population codes as follows: NZ = South Island, New Zealand; FP = all French Polynesian islands; TAH = Tahiti, MOO = Mo’orea; HUA = Huahine; RAI = Raiatea; MAU = Maupiti.

| **Gene stable ID** | **Gene description** | **Gene start (bp)** | **Gene end (bp)** | **Chromosome** | **Gene name** | **NZ vs.** |
| --- | --- | --- | --- | --- | --- | --- |
| ENSTGUG00000006887 | dystrophin | 9154708 | 10148444 | 1 | DMD | HUA |
| ENSTGUG00000010796 |  | 35582832 | 35585680 | 1 |  | HUA |
| ENSTGUG00000010849 | KDEL motif containing 1 | 37962335 | 37970867 | 1 | KDELC1 | RAI |
| ENSTGUG00000011664 |  | 49704389 | 49861236 | 1 |  | MOO |
| ENSTGUG00000011735 | FRY microtubule binding protein | 50626080 | 50796176 | 1 | FRY | HUA |
| ENSTGUG00000011967 | FRAS1 related extracellular matrix protein 2 | 53819513 | 53939566 | 1 | FREM2 | HUA |
| ENSTGUG00000012117 | Taeniopygia guttata SPRY domain containing 7 (SPRYD7), mRNA | 55948726 | 55958266 | 1 | SPRYD7 | MOO |
| ENSTGUG00000012422 | mitochondrial translation release factor 1 | 59579943 | 59591090 | 1 | MTRF1 | HUA |
| ENSTGUG00000012549 |  | 69793105 | 69992841 | 1 |  | MOO |
| ENSTGUG00000013001 | asparaginyl-tRNA synthetase 2, mitochondrial | 86711122 | 86758565 | 1 | NARS2 | TAH |
| ENSTGUG00000013496 | EPH receptor A6 | 101653625 | 102054107 | 1 | EPHA6 | RAI |
| ENSTGUG00000000764 | zinc finger MYND-type containing 11 | 10897250 | 11003676 | 2 | ZMYND11 | HUA |
| ENSTGUG00000001094 | mohawk homeobox | 17223251 | 17392565 | 2 | MKX | HUA |
| ENSTGUG00000001311 | cubilin | 21974685 | 22109468 | 2 | CUBN | RAI |
| ENSTGUG00000001501 | VPS50, EARP/GARPII complex subunit | 25251229 | 25335000 | 2 | VPS50 | MAU |
| ENSTGUG00000004189 | NIMA related kinase 11 | 62221688 | 62308845 | 2 | NEK11 | HUA |
| ENSTGUG00000004894 | transglutaminase 4 | 64731646 | 64741764 | 2 | TGM4 | MOO |
| ENSTGUG00000007906 | von Willebrand factor C domain containing 2 | 85435608 | 85489383 | 2 | VWC2 | HUA |
| ENSTGUG00000008631 |  | 93799185 | 93925718 | 2 |  | HUA |
| ENSTGUG00000010074 |  | 106598017 | 106623803 | 2 |  | RAI |
| ENSTGUG00000011815 | Taeniopygia guttata 2,4-dienoyl-CoA reductase 1 (DECR1), mRNA | 130703700 | 130717968 | 2 | DECR1 | HUA |
| ENSTGUG00000011869 | pyruvate dehyrogenase phosphatase catalytic subunit 1 | 132302035 | 132307232 | 2 | PDP1 | HUA |
| ENSTGUG00000011966 | phosphatidylserine synthase 1 | 133397397 | 133422104 | 2 | PTDSS1 | HUA |
| ENSTGUG00000011987 | lysosomal protein transmembrane 4 beta | 133895664 | 133963069 | 2 | LAPTM4B | HUA |
| ENSTGUG00000011992 | matrilin 2 | 134004617 | 134048324 | 2 | MATN2 | HUA |
| ENSTGUG00000012027 | odd-skipped related transciption factor 2 | 134428506 | 134431204 | 2 | OSR2 | HUA |
| ENSTGUG00000012030 | vacuolar protein sorting 13 homolog B | 134494587 | 134931563 | 2 | VPS13B | HUA |
| ENSTGUG00000012148 | collagen triple helix repeat containing 1 | 136194280 | 136200691 | 2 | CTHRC1 | HUA |
| ENSTGUG00000003612 | pleckstrin homology, MyTH4 and FERM domain containing H2 | 16425219 | 16471280 | 3 | PLEKHH2 | MAU |
| ENSTGUG00000010902 | ARFGEF family member 3 | 50723474 | 50824543 | 3 | ARFGEF3 | TAH |
| ENSTGUG00000011615 | monooxygenase DBH like 1 | 59131319 | 59181152 | 3 | MOXD1 | HUA |
| ENSTGUG00000011773 |  | 61861362 | 61921523 | 3 |  | HUA |
| ENSTGUG00000012218 | blood vessel epicardial substance | 71122911 | 71252155 | 3 | BVES | HUA |
| ENSTGUG00000013229 | phospholipase A2 group VII | 108349385 | 108357841 | 3 | PLA2G7 | HUA |
| ENSTGUG00000002514 |  | 10348610 | 10684047 | 4 |  | HUA |
| ENSTGUG00000004549 |  | 25456400 | 25591830 | 4 |  | HUA |
| ENSTGUG00000005716 | catalase | 6271419 | 6285186 | 5 | CAT | MOO |
| ENSTGUG00000005980 | lysine demethylase 2A | 6538590 | 6567985 | 5 | KDM2A | HUA |
| ENSTGUG00000006012 | G protein-coupled receptor kinase 2 | 6571621 | 6577897 | 5 | GRK2 | HUA |
| ENSTGUG00000006094 | ankyrin repeat domain 13D | 6582669 | 6585026 | 5 | ANKRD13D | HUA |
| ENSTGUG00000008025 |  | 8537039 | 8729742 | 5 |  | HUA |
| ENSTGUG00000009146 | nucleosome assembly protein 1 like 4 | 12881930 | 13003509 | 5 | NAP1L4 | HUA |
| ENSTGUG00000012373 | thyroid stimulating hormone receptor | 41137345 | 41175645 | 5 | TSHR | HUA |
| ENSTGUG00000012820 | REST corepressor 1 | 51054854 | 51135675 | 5 | RCOR1 | HUA |
| ENSTGUG00000012959 | protein phosphatase 2 regulatory subunit B'epsilon | 55239178 | 55306635 | 5 | PPP2R5E | HUA |
| ENSTGUG00000013194 | valosin containing protein lysine methyltransferase | 60520695 | 60523997 | 5 | VCPKMT | HUA |
| ENSTGUG00000004487 | C-type lectin domain family 2 member B-like | 3175115 | 3314084 | 6 |  | HUA |
| ENSTGUG00000004592 | phosphatase domain containing paladin 1 | 3250009 | 3269651 | 6 | PALD1 | HUA |
| ENSTGUG00000007660 | SEC31 homolog B, COPII coat complex component | 16443466 | 16474660 | 6 | SEC31B | MOO |
| ENSTGUG00000007917 |  | 16864802 | 16865718 | 6 |  | MOO |
| ENSTGUG00000003583 |  | 2408013 | 2681518 | 7 |  | HUA |
| ENSTGUG00000004748 | contactin associated protein like 5 | 7834568 | 8039714 | 7 | CNTNAP5 | RAI |
| ENSTGUG00000007152 |  | 13113900 | 13170539 | 7 |  | HUA, TAH |
| ENSTGUG00000007262 | cysteine and serine rich nuclear protein 3 | 13252086 | 13272166 | 7 | CSRNP3 | MOO |
| ENSTGUG00000007583 | ceramide synthase 6 | 13965237 | 14081484 | 7 | CERS6 | HUA |
| ENSTGUG00000010653 |  | 23648780 | 23744147 | 7 |  | MAU |
| ENSTGUG00000011260 | lanosterol synthase | 28607527 | 28615834 | 7 | LSS | HUA, MOO |
| ENSTGUG00000011322 |  | 28963908 | 29034843 | 7 |  | HUA, MAU, TAH |
| ENSTGUG00000011444 | carbamoyl-phosphate synthase 1 | 29360824 | 29444139 | 7 | CPS1 | MAU |
| ENSTGUG00000003826 |  | 2916739 | 2967087 | 8 |  | HUA |
| ENSTGUG00000018373 | zinc finger and BTB domain containing 37 | 3334159 | 3339163 | 8 | ZBTB37 | HUA |
| ENSTGUG00000005495 | dihydropyrimidine dehydrogenase | 8944758 | 9180702 | 8 | DPYD | MAU |
| ENSTGUG00000006332 | Taeniopygia guttata selenoprotein F (SELENOF), mRNA | 12485119 | 12513156 | 8 | SELENOF | RAI |
| ENSTGUG00000006382 |  | 12638237 | 12741483 | 8 |  | HUA |
| ENSTGUG00000007625 | ring finger protein 220 | 17464143 | 17689061 | 8 | RNF220 | HUA |
| ENSTGUG00000009063 | zyg-11 family member A, cell cycle regulator | 22081785 | 22093628 | 8 | ZYG11A | MAU |
| ENSTGUG00000009700 | hook microtubule tethering protein 1 | 24544351 | 24566662 | 8 |  | MAU |
| ENSTGUG00000009745 | nuclear factor I A | 24945852 | 25164582 | 8 | NFIA | HUA, MAU |
| ENSTGUG00000005665 | heparan sulfate 6-O-sulfotransferase 1 | 3776452 | 3976275 | 9 | HS6ST1 | MAU |
| ENSTGUG00000007084 | protein phosphatase 2 regulatory subunit B''alpha | 6573476 | 6609786 | 9 | PPP2R3A | RAI |
| ENSTGUG00000008270 |  | 10768020 | 10786905 | 9 |  | RAI |
| ENSTGUG00000009820 |  | 17638634 | 17660277 | 9 |  | HUA |
| ENSTGUG00000010675 | G protein subunit beta 4 | 20069493 | 20079705 | 9 | GNB4 | HUA |
| ENSTGUG00000006654 | myosin VA | 8523659 | 8598997 | 10 | MYO5A | HUA |
| ENSTGUG00000008476 | solute carrier organic anion transporter family member 3A1 | 14621964 | 14731878 | 10 | SLCO3A1 | MOO |
| ENSTGUG00000009006 |  | 18257909 | 18275693 | 10 |  | HUA |
| ENSTGUG00000017715 | microRNA 184 | 20485775 | 20485848 | 10 | MIR184 | HUA |
| ENSTGUG00000010006 | zinc finger protein 710 | 20492147 | 20493497 | 10 | ZNF710 | HUA |
| ENSTGUG00000010009 | semaphorin 4B | 20501862 | 20505986 | 10 | SEMA4B | HUA |
| ENSTGUG00000005417 | E2F transcription factor 4 | 4815708 | 4825887 | 11 | E2F4 | HUA, MOO, RAI |
| ENSTGUG00000005428 | engulfment and cell motility 3 | 4827918 | 4834507 | 11 | ELMO3 | HUA, MOO, RAI |
| ENSTGUG00000005766 | tubulin polymerization promoting protein family member 3 | 5056834 | 5058661 | 11 | TPPP3 | HUA |
| ENSTGUG00000008994 | URI1, prefoldin like chaperone | 14711166 | 14741310 | 11 |  | MAU |
| ENSTGUG00000008404 | coiled-coil domain containing 174 | 11785828 | 11797268 | 12 | CCDC174 | MAU |
| ENSTGUG00000010102 | contactin 4 | 19614606 | 19766886 | 12 |  | HUA |
| ENSTGUG00000000209 | interleukin 12B | 1338072 | 1344091 | 13 | IL12B | HUA |
| ENSTGUG00000000776 | FAT atypical cadherin 2 | 6519325 | 6562614 | 13 | FAT2 | RAI |
| ENSTGUG00000000816 |  | 6633705 | 6783751 | 13 |  | HUA |
| ENSTGUG00000001246 | core histone macro-H2A.1 | 9525620 | 9569022 | 13 | H2AFY | HUA |
| ENSTGUG00000004540 | KIAA0556 | 3169643 | 3207271 | 14 | KIAA0556 | HUA |
| ENSTGUG00000008135 |  | 12554331 | 12555206 | 14 |  | MAU |
| ENSTGUG00000009821 |  | 11316478 | 11424936 | 15 |  | MOO |
| ENSTGUG00000009830 |  | 11366499 | 11374215 | 15 |  | MOO |
| ENSTGUG00000009905 | sodium/glucose cotransporter 1 | 11507233 | 11538464 | 15 |  | HUA |
| ENSTGUG00000003398 |  | 5138627 | 5267364 | 17 |  | MAU |
| ENSTGUG00000003550 |  | 5370218 | 5372236 | 17 |  | MAU |
| ENSTGUG00000003552 | dolichyl-phosphate mannosyltransferase subunit 2, regulatory | 5374125 | 5374580 | 17 | DPM2 | MAU |
| ENSTGUG00000003554 | ST6 N-acetylgalactosaminide alpha-2,6-sialyltransferase 4 | 5376833 | 5488169 | 17 | ST6GALNAC4 | HUA, MAU, TAH |
| ENSTGUG00000003556 |  | 5380700 | 5381723 | 17 |  | MAU |
| ENSTGUG00000003559 | adenylate kinase 1 | 5383752 | 5387428 | 17 | AK1 | MAU |
| ENSTGUG00000003704 | TruB pseudouridine synthase family member 2 | 5499738 | 5503313 | 17 | TRUB2 | HUA, TAH |
| ENSTGUG00000007364 | PBX homeobox 3 | 10940614 | 11046375 | 17 | PBX3 | HUA |
| ENSTGUG00000007428 | Ral GEF with PH domain and SH3 binding motif 1 | 11515416 | 11577459 | 17 | RALGPS1 | MAU |
| ENSTGUG00000003385 | transmembrane channel like 6 | 1691137 | 1696099 | 18 | TMC6 | MAU |
| ENSTGUG00000003410 | hepatocyte growth factor-regulated tyrosine kinase substrate | 1699283 | 1707780 | 18 | HGS | MAU |
| ENSTGUG00000003446 | mitochondrial ribosomal protein L12 | 1708980 | 1712466 | 18 | MRPL12 | MAU |
| ENSTGUG00000006238 | myosin-3 | 5544437 | 5559002 | 18 |  | MAU |
| ENSTGUG00000006545 |  | 5571232 | 5573803 | 18 |  | MAU |
| ENSTGUG00000006551 | myosin heavy chain, skeletal muscle, adult | 5585671 | 5600256 | 18 |  | MAU |
| ENSTGUG00000007675 | chromosome 18 open reading frame, human C17orf62 | 6697273 | 6703781 | 18 | CYBC1 | HUA |
| ENSTGUG00000007809 | jumonji domain containing 6, arginine demethylase and lysine hydroxylase | 7731908 | 7741193 | 18 | JMJD6 | HUA |
| ENSTGUG00000007819 | ST6 N-acetylgalactosaminide alpha-2,6-sialyltransferase 2 | 7751042 | 7828425 | 18 | ST6GALNAC2 | HUA |
| ENSTGUG00000007923 | ubiquitin conjugating enzyme E2 O | 7960889 | 7981633 | 18 | UBE2O | MAU |
| ENSTGUG00000008958 |  | 11474946 | 11478895 | 19 |  | HUA, MOO |
| ENSTGUG00000008962 |  | 11481381 | 11485166 | 19 |  | HUA, MOO |
| ENSTGUG00000008966 | PITPNM family member 3 | 11496529 | 11534638 | 19 | PITPNM3 | HUA, MOO |
| ENSTGUG00000002039 | zinc finger and BTB domain containing 17 | 109799 | 116290 | 21 | ZBTB17 | TAH |
| ENSTGUG00000004728 |  | 2773733 | 2777396 | 22 |  | HUA |
| ENSTGUG00000004741 | ASH2 like, histone lysine methyltransferase complex subunit | 2784936 | 2792494 | 22 | ASH2L | HUA |
| ENSTGUG00000001038 |  | 2978316 | 2979139 | 23 |  | HUA |
| ENSTGUG00000001039 |  | 2987758 | 2989220 | 23 |  | HUA |
| ENSTGUG00000001130 | runt related transcription factor 3 | 3280382 | 3312215 | 23 | RUNX3 | HUA |
| ENSTGUG00000001160 | myomesin 3 | 3534172 | 3567527 | 23 | MYOM3 | HUA |
| ENSTGUG00000001176 | fatty acid-binding protein, liver-like | 3568981 | 3570931 | 23 |  | HUA |
| ENSTGUG00000001265 | hes related family bHLH transcription factor with YRPW motif-like | 3743972 | 3744625 | 23 | HEYL | HUA |
| ENSTGUG00000001266 | poly(A) binding protein cytoplasmic 4 | 3755982 | 3768257 | 23 | PABPC4 | HUA |
| ENSTGUG00000001438 | leucine zipper protein 1 | 4294754 | 4297891 | 23 | LUZP1 | HUA |
| ENSTGUG00000001329 |  | 2526723 | 2529254 | 26 |  | MAU |
| ENSTGUG00000001330 | troponin T2, cardiac type | 2537936 | 2542499 | 26 | TNNT2 | MAU |
| ENSTGUG00000001345 | transmembrane protein 9 | 2697635 | 2701249 | 26 | TMEM9 | HUA |
| ENSTGUG00000001363 | PTPRF interacting protein alpha 4 | 2749706 | 2780727 | 26 | PPFIA4 | MOO |
| ENSTGUG00000001381 | adenosine receptor A1 | 2801182 | 2823090 | 26 | ADORA1 | HUA |
| ENSTGUG00000001511 | plexin A2 | 3571959 | 3682353 | 26 | PLXNA2 | HUA |
| ENSTGUG00000002482 |  | 2319216 | 2319366 | 27 |  | HUA |
| ENSTGUG00000002515 |  | 2388654 | 2627252 | 27 |  | HUA |
| ENSTGUG00000002656 | vacuolar protein sorting 25 homolog | 2509069 | 2631604 | 27 | VPS25 | HUA |
| ENSTGUG00000002727 | tubulin gamma 1 | 2596517 | 2601876 | 27 | TUBG1 | HUA |
| ENSTGUG00000002735 | contactin associated protein 1 | 2609963 | 2615943 | 27 | CNTNAP1 | HUA |
| ENSTGUG00000002970 |  | 2944147 | 2950604 | 27 |  | HUA |
| ENSTGUG00000000551 |  | 2758875 | 2784928 | 28 |  | MAU |
| ENSTGUG00000000664 | WD repeat domain 18 | 3047464 | 3054358 | 28 | WDR18 | RAI |
| ENSTGUG00000000667 |  | 3061236 | 3072627 | 28 |  | RAI |
| ENSTGUG00000002908 | LHFPL tetraspan subfamily member 3 | 12508602 | 12668585 | 1A | LHFPL3 | RAI |
| ENSTGUG00000007680 | otogelin like | 39135461 | 39219032 | 1A | OTOGL | MAU |
| ENSTGUG00000012520 | adenosine deaminase 2 | 70536304 | 70554685 | 1A | ADA2 | TAH |
| ENSTGUG00000005324 | chloride voltage-gated channel 5 | 16132112 | 16150679 | 4A | CLCN5 | HUA, RAI |

**Table S3:** Outlier SNPs identified using *PCAdapt* and within 10kb of genes thought to be associated with bill/body size differentiation. Note: no candidate genes identified from New Zealand (SNZ) vs. Tahiti (TAH) comparison. Outlier SNP distances based on the *T. guttata* genome assembly.

| **SNP Position** | **Genes within 10kb** | **Distance (bp)** | **Justification** | **Source(s)** | **Comparison** |
| --- | --- | --- | --- | --- | --- |
| Chr11: 4824624 | *E2F4* | In gene | Associated with craniofacial defects in mice | Humbert *et al.* (2000) | SNZ vs. MOO |
| Chr11: 4824624 |  | In gene |  |  | SNZ vs. HUA |
| Chr11: 4824624 |  | In gene |  |  | SNZ vs. RAI |
| Chr1: 53939473 | *FREM2* | In gene | Associated with craniofacial variation in European glass eels | De Meyer *et al.* (2017) | SNZ vs. MOO |
| Chr8: 25070276 | *NFIA* | In gene | Associated with bill length in the house sparrow | Rao *et al.* (2014); | SNZ vs. HUA |
| Chr8: 25070276 |  | In gene |  | Lundregan *et al.* (2018) | SNZ vs. MAU |
| Chr8: 25128841 |  | In gene |  |  | SNZ vs. HUA |
| Chr2: 134435555 | *OSR2* | 4,351 | Demonstrated to play a role in beak development in birds and craniofacial defects in mice | Brugmann *et al.* (2010); | SNZ vs. HUA |
| Chr2: 134435642 |  | 4,438 |  | Stanier & Moore (2004) | SNZ vs. HUA |
| Chr17: 11049402 | *PBX3* | 3,027 | Associated with craniofacial shape in mice | Amaya (2015) | SNZ vs. HUA |
| Chr17: 11049431 |  | 3,056 |  |  | SNZ vs. HUA |
| Chr2: 133393131 | *PTDSS1* | 4,266 | Under directional selection in birds of paradise | Prost *et al.* (2018) | SNZ vs. HUA |
| Chr17: 11554371 | *RALGPS1* | In gene | Associated with craniofacial shape in mice | Amaya (2015) | SNZ vs. MAU |
| Chr18: 1702196 | *TMC6* | 6,097 | Associated with craniofacial variation in baboons | Joganic (2016) | SNZ vs. MAU |
| Chr2: 134865989 | *VPS13B* | In gene | Under directional selection between species of Darwin’s finches | Lawson & Petren (2017) | SNZ vs. HUA |
| Chr2: 25310237 | *VPS50* | In gene | Associated with bill length in Berthelot’s pipit | Armstrong *et al.* (2018) | SNZ vs. MAU |
| Chr2: 10960228 | *ZMYND11* | In gene | Associated with craniofacial disorders in humans | Cobben *et al.* (2014) | SNZ vs. HUA |
| Chr2: 10960308 |  | In gene |  |  | SNZ vs. HUA |

**Table S4:** SNPs with PIP ≥ 0.5 in BSLMM analysis for PC1.

| **SNP Position** | **PIP** | **Genes within 10kb** | **Distance (bp)** | **Candidate?** | **Justification** | **Source(s)** |
| --- | --- | --- | --- | --- | --- | --- |
| Chr 5: 51019952 | 0.21 | - | - | - | - | - |
| Chr 7: 10776038 | 0.208 | *OBSL1* | 6428 | Yes | Cytoskeletal adapter protein associated with body size in humans and cetaceans | Hanson et al. (2009); Sun et al. (2019) |
|  |  | *INHA* | 1341 | No | - | - |
| Chr 14: 10466122 | 0.13 | *TOM1L2* | In gene | No | - | - |
| Chr 10: 17233572 | 0.097 | *IGF1R* | In gene | Yes | Transmembrane receptor of insulin-like growth factor 1 (IGF1). This pathway has a well-known role in body size scaling in many taxa and bill size in black-bellied seedcracker | Beccavin et al. (2001); Beckman et al. (2003); Grossi et al. (2015); Sparkman et al. (2010); Sutter et al. (2007); vonHoldt et al. (2018) |
| Chr 1A: 45484302 | 0.082 | *-* | - | - | - | - |
| Chr 10: 16547610 | 0.078 | *-* | - | - | - | - |
| Chr 15: 12584727 | 0.077 | *NOS1* | In gene | No | - | - |
| Chr 11: 9389563 | 0.077 | *-* | - | - | - | - |
| Chr 23: 3298568 | 0.076 | *LDLRAP1* | In gene | No | - | - |
|  |  | *RUNX3* | In gene | Yes | Modulates Bone Morphogenetic Protein (BMP) signaling during craniofacial development in zebrafish. BMP4 is strongly associated with morphological variation of beaks in Darwin's finches | Dalcq et al. (2012); Abzhanov (2004) |
| Chr 1: 70693444 | 0.076 | *-* | - | - | - | - |
| Chr 6: 6929772 | 0.076 | *-* | - | - | - | - |
| Chr 2: 24089154 | 0.075 | *CDK14* | In gene | Yes | Associated with beak morphology in Darwin’s finches | Lawson and Petren (2017) |
| Chr 2: 130773479 | 0.075 | *-* | - | - | - | - |
| Chr 6: 30042357 | 0.074 | *-* | - | - | - | - |
| Chr 12: 14253180 | 0.074 | *ATXN7* | In gene | No | - | - |
| Chr 2: 10960228 | 0.074 | *ZMYND11* | In gene | Yes | Associated with craniofacial disorders in humans | Cobben *et al.* (2014) |
| Chr 14: 10466121 | 0.069 | *TOM1L2* | In gene | No | - | - |
| Chr 4: 9274516 | 0.068 | *INPP4B* | In gene | Yes | Associated with skull shape in the house mouse. | Amaya (2015); |
|  |  |  |  |  |  | Pallares et al. (2015) |
| Chr 1: 43969483 | 0.063 | *-* | - | - | - | - |
| Chr 2: 121483640 | 0.063 | *SGK3* | 2809 | No | - | - |
| Chr 3: 61227508 | 0.059 | *THEMIS* | In gene | No | - | - |
| Chr 4: 29818610 | 0.055 | *FNIP2* | 6879 | No | - | - |
| Chr 5: 4838538 | 0.052 | - | - | - | - | - |
| Chr 14: 14065409 | 0.051 | - | - | - | - | - |

**
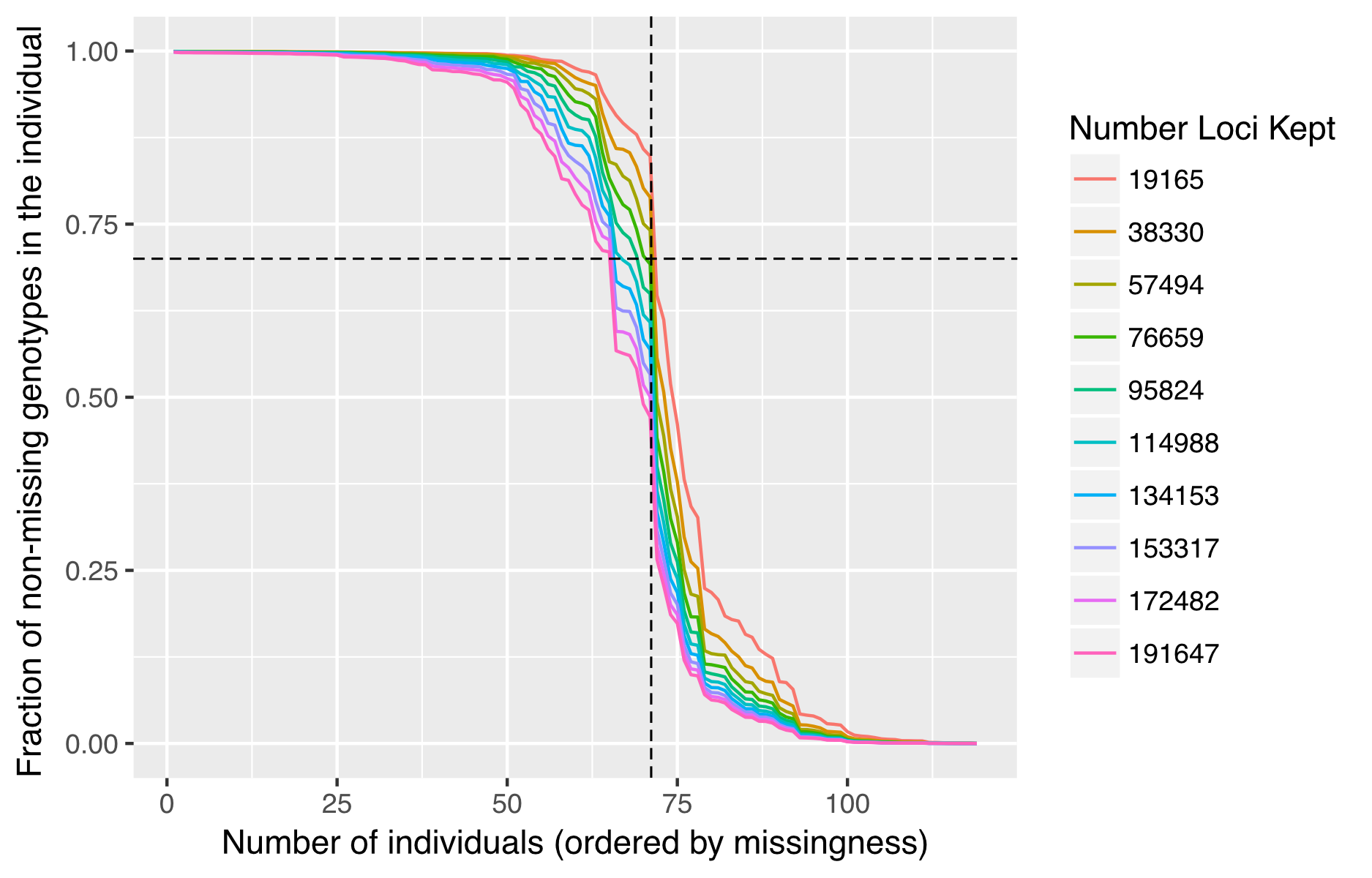
**

**Figure S1:** Missingness plot generated using *genoscapeRtools* used to determine the optimal number of samples and SNPs to retain for downstream analyses. Dashed lines indicate individuals retained and missingness filters applied.
